# Supplementary material for: Coherent X-rays reveal the influence of cage effects on ultrafast water dynamics
Source: Nat Commun. 2018 May 15;9:1917. doi: 10.1038/s41467-018-04330-5 (PMC5953967; doi:10.1038/s41467-018-04330-5)
Supplement: Supplementary file 2 — Supplementary Information [file 41467_2018_4330_MOESM2_ESM.pdf]

SUPPLEMENTARY INFORMATION

Coherent X-rays reveal the influence of cage effects on ultrafast water dynamics

Fivos Perakis[1,2,*], Gaia Camisasca[1], Thomas J. Lane[2], Alexander Späh[1], Kjartan Thor Wikfeldt[1], Jonas A. Selberg[3], Felix Lehmkühler[4,5], Harshad Pathak[1], Kyung Hwan Kim[1], Katrin Amann-Winkel[1], Simon Schreck[1], Sanghoon Song[2], Takahiro Sato[2], Marcin Sikorski[2,6], Andre Eilert[2], Trevor McQueen[2], Hirohito Ogasawara[2], Dennis Nordlund[2], Wojciech Roseker[4,5], Jake Koralek[2], Silke Nelson[2], Philip Hart[2], Roberto Alonso-Mori[2], Yiping Feng[2], Diling Zhu[2], Aymeric Robert[2], Gerhard Grübel[4,5], Lars G. M. Pettersson[1], and Anders Nilsson[1,**]

[1] *Department of Physics, AlbaNova University Center, Stockholm University, S-106 91 Stockholm, Sweden,*

[2] *SLAC National Accelerator Laboratory, 2575 Sand Hill Road, Menlo Park, California 94025,*

[3] *Biomedical and X-ray Physics, Department of Applied Physics, AlbaNova University Center, KTH Royal Institute of Technology, S-10691 Stockholm, Sweden*

[4] *Deutsches Elektronen-Synchrotron DESY, Notkestr. 85, 22607 Hamburg, Germany,*

[5] *Hamburg Centre for Ultrafast Imaging, Luruper Chaussee 149, 22761 Hamburg, Germany*

[6] *European XFEL, Holzkoppel 4, 22869 Schenefeld, Germany*

* f.perakis@fysik.su.se

** andersn@fysik.su.se

Supplementary Notes

**1.**	Contrast estimation metrics	2
**2.**	Fluence dependence	3
**3.**	Ionization probability	4
**4.**	FEL shot filtering	6
**5.**	Mean photon density per δt	8
**6.**	Signal to noise estimation	9
**7.**	Maximum contrast analytical estimation	10
**8**	Thermal velocity model	13

Supplementary Note 1: Contrast estimation metrics

In order to cross-validate the analytical estimator metric, we analysed the data by using an alternative approach: the maximum likelihood estimation. In this case, the contrast was not estimated on a single shot basis, but instead the shots were sorted by the mean photon density $\bar{k}$, which is the average number of photons/pixel. For each shot the photon histogram was estimated, and then the histogram of shots with the same $\bar{k}$ were averaged, where for the binning a spacing of 0.001 photons/pixel/shots was used. The contrast was estimated by fitting the averaged histogram using equation (1) with the maximum likelihood estimation and is plotted as function of $\bar{k}$ in Supplementary Fig. 1a. The mean contrast was estimated by a weighted average, with weights as the number of shots in each bin, and is shown as the dashed green line. In this case, the average is $\beta = 0.069$ and the standard error is 0.002.

This approach is potentially more general, since it utilizes the whole photon histogram k, and can thereby be used in the case where not only 1 and 2 photon events are recorded, but also higher counts. The disadvantage is that it cannot be performed on a single-shot basis, but requires averaging of more shots with the same mean photon density. By analysing the same dataset with the analytical estimator, the contrast is estimated on a single-shot basis and the cumulative average is shown in Supplementary Fig. 1b. In this case, the average is $\beta = 0.067$ and the standard error is 0.002, which agrees within the signal-to-noise with the contrast value obtained by the maximum likelihood estimation approach. Here we emphasize that not only two different estimators were used for this comparison, but also two different ways of averaging the data: in the former case sorted by mean photon density (maximum likelihood estimation) and in the latter on a single-shot basis (analytical estimator).

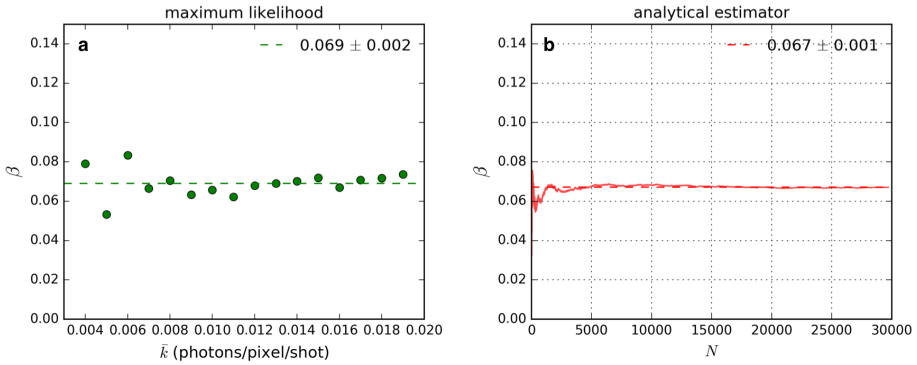

Supplementary Figure 1 | Estimator comparison. a, Contrast β calculated using the maximum likelihood estimator as a function of mean photon density in photons/pixel/shot units. The dashed line indicates the weighted average and the legend shows the mean value plus minus the standard error. **b,** Cumulative average of the contrast β estimated using the analytical estimator on a single-shot basis. The dashed line is the average value, as indicated in the legend plus minus the standard error.

The reason that the data in Supplementary Fig. 1 become dispersed in the low photon density range is because the limited number of shots available in this range. By attenuating the beam one can also obtain with precision the speckle contrast at lower fluence, although a larger number of shots is needed.

Supplementary Note 2: Fluence dependence

As discussed in the main text in order to estimate any possible beam induced heating effects, that can arise within the pulse duration we have analysed the *Q*-position of the first diffraction peak as a function of photon density. This analysis complements the analysis discussed at Supplementary Fig. 1a, where the contrast is also analysed as a function of the photon density. In Supplementary Fig. 1a we concluded that we do not observed any beam induced changes in the estimated contrast, as the contrast is constant for a broad range of photon densities, that corresponds to different pulse intensities. Here in addition, we perform as similar analysis for the longer pulse duration that was used δt = 120 fs and for two different temperatures, T = 296 K and T = 328 K, shown in Supplementary Fig. 2. The y-range chosen matches Fig. 4b of the main manuscript and the x axis is the photon counts of the ePIX detector (in logarithmic scale), as shown in Supplementary Fig. 1a, which is proportional to the incident energy. If there was a temperature rise occurring within the pulse duration, one would expect the *Q* to shift at higher values with increasing fluence, whereas here the *Q* position of the first diffraction maximum in *I(Q)* appears independent of the fluence over an of magnitude.

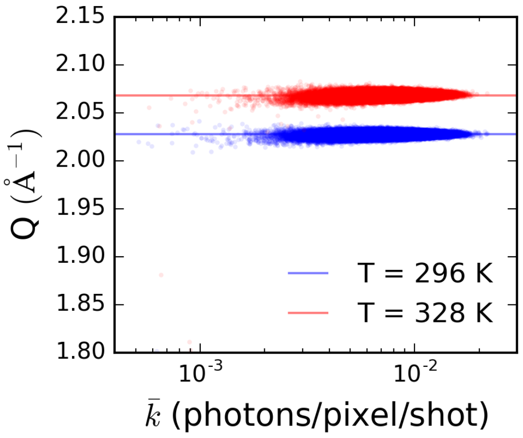

Supplementary Figure 2 | The Q position of the first diffraction maximum of the angularly integrated intensity I(Q) as a function of photon density $\bar{k}$ for δt = 120 fs.

Supplementary Note 3: Ionization probability

In order to estimate the magnitude of possible contributions due to ionization events that can occur during the pulse duration, we estimate the ionization probability as a function of fluence[1].

The number of molecules N_{mol} in the probed volume can be estimated by taking into account the number density of water ($n = 33.3679 \cdot 10^{27}\ \mathrm{m}^3$) as well as the focus size (radius r = 1 μm) and sample thickness (*w* = 98 μm). This yields:

$$N_{mol} = n \cdot V = n \cdot (w \cdot \pi r^2) \approx 1.03 \cdot 10^{13}\text{molecules}$$

The total number of absorption events is proportional to the number of photons per unit area I_0 and the sample thickness w, where the proportionality constant is the absorption coefficient μ ($\mu \approx 1\ \mathrm{mm}^{-1}$ at 8.2 keV for water): This is also the number of photoelectrons emitted:

$$N_e = I_0 \cdot w \cdot \mu$$

The I_0 is defined by the ratio of incident energy ($\approx 10\ \mu\mathrm{J}$) over the area $\pi \mathrm{r}^2$, which is in the order of 10^9 photons/$\mu\mathrm{m}^2$. The ionization probability per $\mu\mathrm{m}^2$ is defined as the number of photoelectrons over the number of molecules in the probed volume:

$$P_{ionization} = \frac{N_e}{N_{mol}}$$

The ionization probability as a function of fluence is shown in Supplementary Fig. 3.

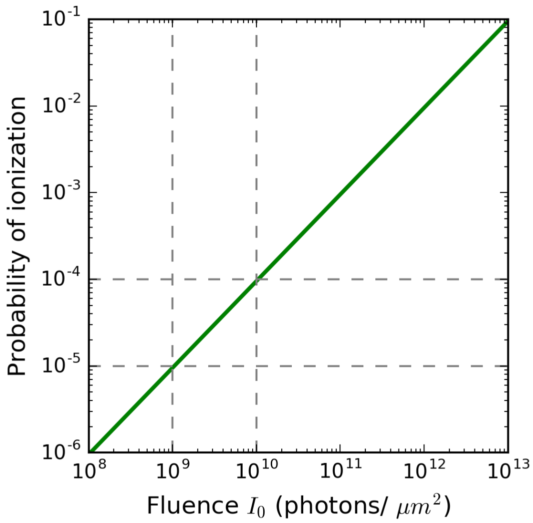

Supplementary Figure 3 | The probability of ionization as a function of fluence. For the current experimental conditions the fluence is estimated between 10^9 to 10^{10} photons/pulse resulting 10^{-5} to 10^{-4} ionization events per molecule per $\mu\mathrm{m}^2$.

For fluences of the order of 10^9 to 10^{10} photons/pulse the ionization probability is between 10^{-5} to 10^{-4} electrons per molecule, assuming single photon absorption. In other words this corresponds to one ionization event per 10^4 to 10^5 molecules, which is well beyond the sensitivity of the current experiment. At higher fluences however, which can be reached for example by utilizing the full (i.e. pink) beam of LCLS combined with a nanofocus, the ionization probability can become significant [13]. In addition, previous experimental [14] and theoretical [15] investigations indicate that each photoelectron will lead to many secondary electrons depending on the photon energy. However, since the ionization probability depends on the fluence (Supplementary Fig. 3), any potential contributions to the dynamics would also involve fluence dependence. Since the speckle contrast, which reflects the dynamics, (Supplementary Fig. 1) appears fluence-independent, we tentatively conclude that we do not observe any major contributions due to ionization.

Supplementary Note 4: FEL shot filtering

Obtaining the speckle contrast experimentally requires filtering the obtained data according to the following criteria: the X-rays hit the droplets and the recorded X-ray diffraction indicated that the droplet is in the liquid state and not frozen the X-ray pulses were within the desired pulse duration, the pulse intensity was sufficient to estimate the contrast with precision and the photon energy was within a certain range in order to maintain the desired *Q* resolution.

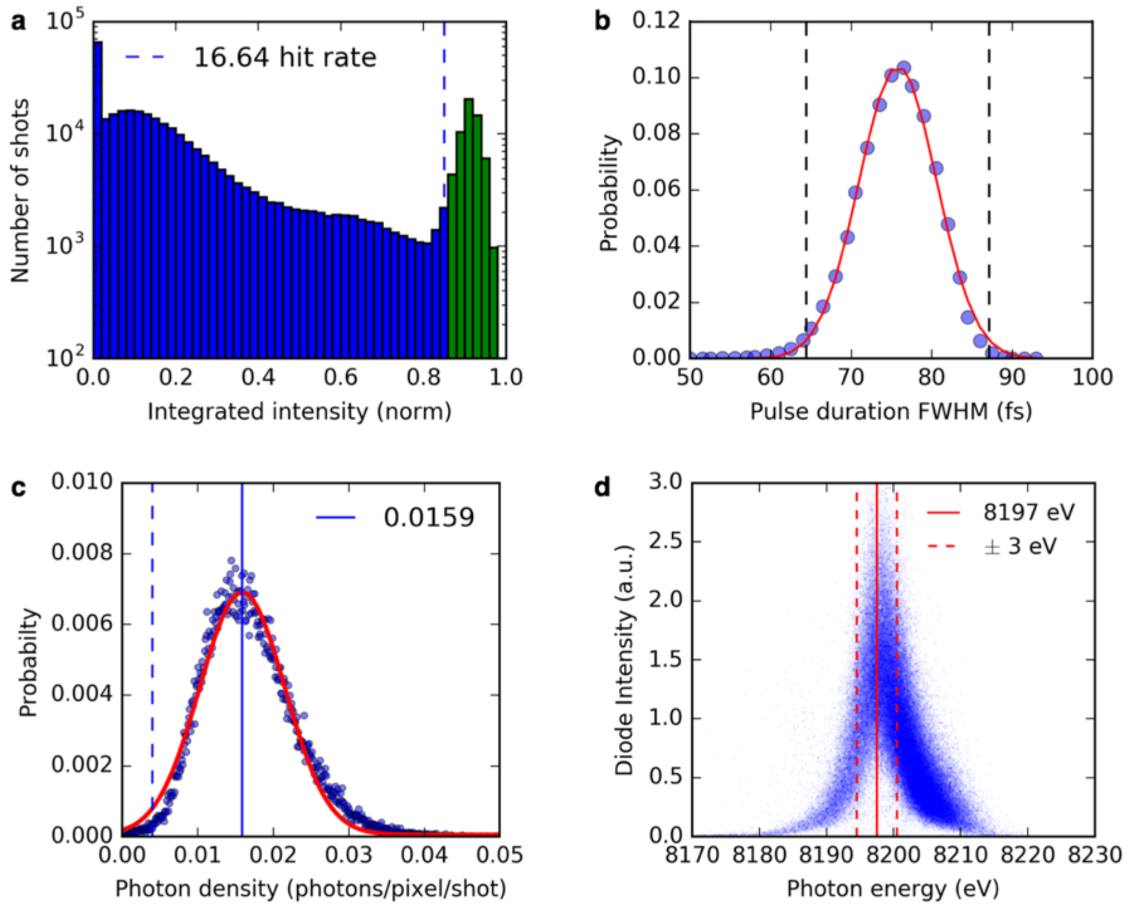

Supplementary Figure 4 | The criteria for filtering shots. a, Histogram of the integrated intensity of the CSPAD after normalization for *T* = 250 K and pulse duration 75 fs. The dashed line indicates the threshold filter. The legend shows the percentage hit rate (%) based on the threshold. **b,** The probability histogram of the pulse duration full-width at half-maximum (FWHM) obtained from the XTCAV. The dashed lines indicate the cut-offs **c,** The photon density distribution obtained from the ePix detector (in units of photons/pixel/shot). The fit corresponds to a Gaussian and the solid line is the mean obtained from the fit (and the corresponding value in the legend in photons/pixel/shot). The dashed line indicates the threshold filter. **d,** The correlation plot between the pulse intensity (obtained by the diode measuring the direct beam) and the photon energy (eV). The solid line is the maximum and the dashed lines indicate the cut offs.

The data from the CSPAD detector[2] were used in order to distinguish between hits and misses, as well as for removing the corresponding ice shots (for the supercooled conditions), similar to previous approaches[3–5]. For each shot the angularly integrated intensity was calculated was normalized at the maximum intensity, as shown in the green region in Supplementary Fig. 4a. In the case of liquid water that is a broad peak and therefore integrating over all momentum transfer Q one would get a high value. On the other hand, in the case of ice the obtained peak is much narrower and the integrated intensity over all Q's gives a lower value. Therefore, by imposing a threshold filter at 0.85, we only include in the dataset the shots over this value and thus eliminate ice shots and misses. The exact value of the threshold filter is not very important, as it does not affect significantly the final results, as long as the peak at higher integrated intensities is separated by the threshold filter.

The data from the XTCAV detector[6] were used to obtain information on the pulse duration of each single shot. The probability histogram is shown in Supplementary Fig. 4b, where the solid red line depicts a Gaussian fit with a mean value of δt = 75.5 fs. The dashed lines at δt ±9.7fs from the mean are the cut-off limits that were chosen, based on two times the FWHM of the distribution. This choice of the cut-off values does not affect significantly the end results and it turned out to be more useful for the datasets where the pulse duration was varied.

The distribution of the mean photon density $\bar{k}$ obtained from the ePix detector[7] is shown in Supplementary Fig. 4c. In this distribution, where shots with fewer photons have lower photon density (in units of photons/pixel), the average value was in the order of 0.01 photons/pixel. For very weak shots below this value, the contrast cannot be calculated with precision using the analytical estimation approach, which relies on the number of one and two-photon counts, similar to previous observations[8]. Therefore, we impose a threshold limit at 0.003 photons/pixel, and only include in the contrast estimation shots above this value.

The final filtering criterion is the energy of the electron bunch giving the X-ray pulse. This approach has been used previously[8,9] and is based on the correlation between the electron energy calibrated to the corresponding X-ray photon energy. Supplementary Fig. 4d shows the overlay between sorted X-ray photon energy before the Si(111) monochromator and the throughput intensity of the X-ray beam after the sample measured by a diode. The vertical solid line is the mean of the distribution and the dashed vertical lines indicate the range that was used for the analysis.

Supplementary Note 5: Mean photon density per δt

The photon density at each pulse duration depends on the LCLS performance at the given pulse duration. Here, we report the mean photon density for different pulse duration for T = 296K, which is obtained by a Gaussian fit of the distribution in each case.

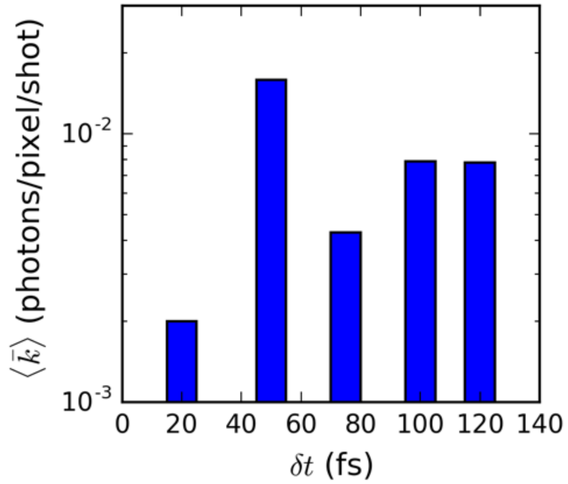

Supplementary Figure 5 | The mean photon density $\langle \bar{k} \rangle$ as a function of pulse duration δt for T = 296 K.

Supplementary Note 6: Signal-to-noise estimation

The required number of shots at different photon densities can be estimated analytically by calculating the signal-to-noise ratio as a function of number of shots[8], by the following expression:

$$\frac{\sigma_\beta}{\beta} = \frac{1}{\bar{k} \cdot \beta} \sqrt{\frac{2(1+\beta)}{n_{pix} \cdot N}}$$

where n_{pix} is the number of pixels and N is the number of shots. To illustrate the scaling of the signal-to-noise ratio with number of shots we estimate the $\frac{\sigma_\beta}{\beta}$ in two different regime: for $\bar{k} = 10^{-2}$ photons/pixel/pulse, relating to the current settings, and for $\bar{k} = 10^{-3}$ photons/pixel/pulse, which could be related the estimated photon density using a split-and-delay that can be used to measure longer timescales.

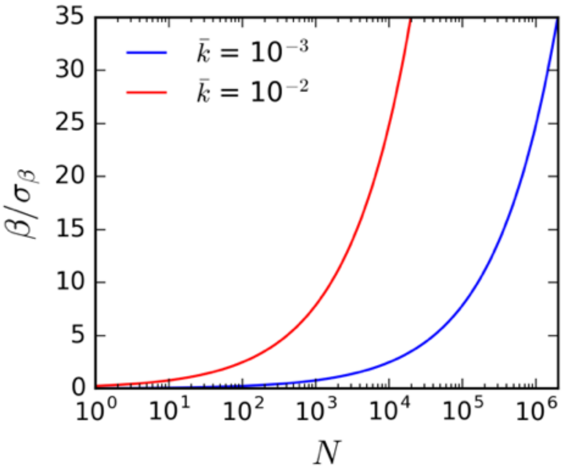

Supplementary Figure 6 | The estimated signal-to-noise ratio as a function of number of shots N for two conditions with photon density $\bar{k}$ (photons/pixel/pulse).

Here, we use the following values of the current experiment $\beta = 0.07$ and n_{pix}= 352×384×2. One can see that, as presented in the main manuscript, for $\bar{k} \approx 10^{-2}$ one can reach a signal-to-noise ratio of 35 (which for $\beta = 0.07$ corresponds to $\sigma_\beta = 0.002$) after approximately 10^4 shots. For lower photon densities $\bar{k} \approx 10^{-3}$ approximately 10^6 shots are required per condition, which is still within the experimental capabilities of current FEL sources. One can also improve this scaling by implementing a larger number of detectors covering a larger fraction of the azimuthal angle, thereby increasing n_{pix}, which will also be possible with the rapid advancements in the detector technology. Finally, by utilizing the higher repetition rate of the upcoming FEL sources one can dramatically improve the signal-to-noise ratio by measuring several orders of magnitude more shots at each experimental condition.

Supplementary Note 7: Maximum contrast analytical estimation

The maximum contrast can be estimated analytically for the given experimental conditions, which can be used to optimize the radial and detector resolution of the experimental settings[10,11]. For scattering in transmission geometry, the radial and detector resolution are given by:

$$M_{rad} = \left[1 + \frac{Q^2(\Delta E/E)^2(s_v{}^2\cos^2\theta + d^2 sin^2\theta)}{4\pi^2}\right]^{1/2} \quad (2)$$

$$M_{det} = \left[1 + \frac{p^4 s_h^2(s_v^2 cos^2 2\theta + d^2 sin^2 2\theta)}{\lambda^4 L^4 M_{rad}^2}\right]^{1/2} \quad (3)$$

where the parameters are defined in Supplementary Table 1. According to the contrast definition $\beta = 1/M$ the maximum contrast for the given parameters will be:

$$\tilde{\beta}_0 = 1/(M_{rad} \cdot M_{det}) \quad (4)$$

In Supplementary Fig. 7 is shown the contrast calculated as a function of momentum transfer Q, sample thickness *d*, sample-detector distance *L* and pixel size *p*. Here each calculation assumes varying one parameter, while keeping all the other constant with values depicted in Supplementary Table 1. In all case the dashed line indicate the contrast value for the current experimental settings, which is estimated to be $\tilde{\beta}_0 = 0.081$. This is slightly higher than the experimentally estimated value of $\beta_0 = 0.069$, which can be attributed to the small aberrations of the X-ray optics, as well as pointing and coherence shot-to-shot fluctuations[12]. From the calculated contrast in Supplementary Fig. 7, we conclude that the increase sample thickness is one of the main limitations of the experimental settings, due to the limited coherence length of the source for wide angle scattering geometries (WAXS). That is evident as well in the high value obtained for the radial resolution M_{rad} = 10.81, contrary to the detector resolution M_{det} = 1.15. This is an intrinsic limitation of the experiment, which arises from the weak cross-section of water in the used energy in combination with the requirement of large detector-sample distance needed to resolve the speckle pattern.

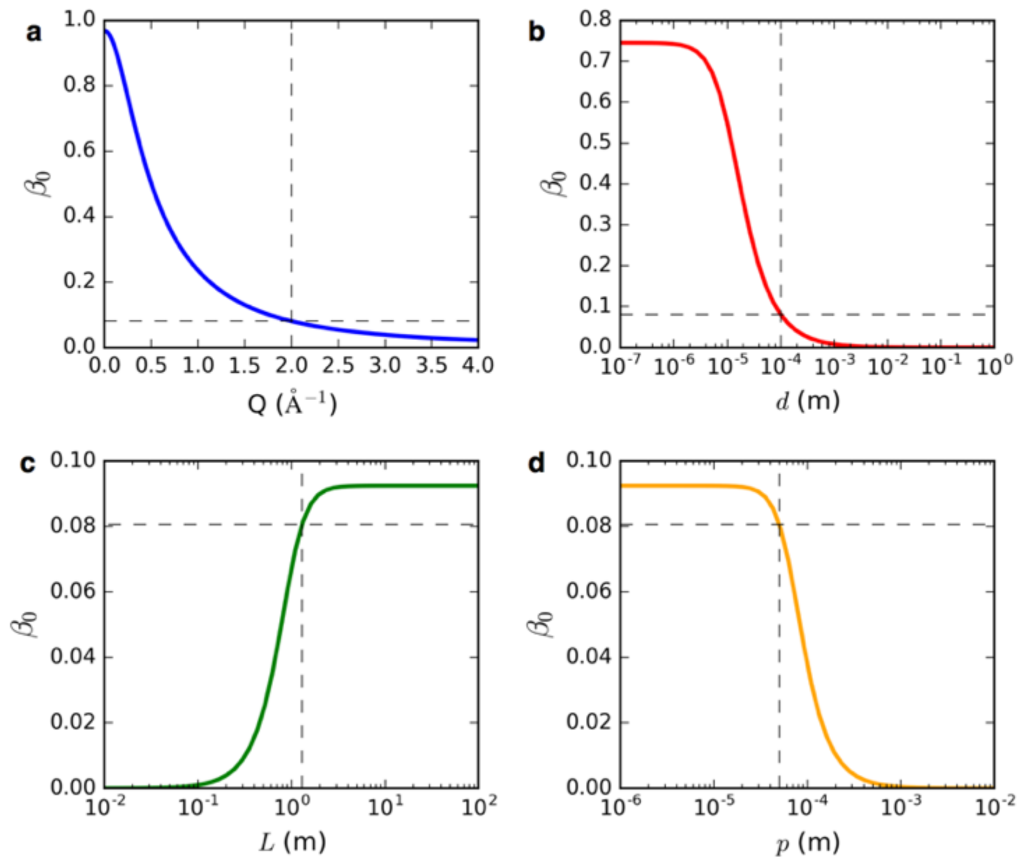

Supplementary Figure 7 | Analytical estimation of the maximum contrast β_0 that can be obtained for the given settings. The contrast β_0 is calculated as a function of momentum transfer Q, (b) sample thickness, (c) sample detector distance L and (d) pixel size *p*. The dashed lines indicate the values for the current experimental settings.

Supplementary Table 1: The parameters used for the analytical calculations and the resulting values

Parameter	Definition	Value
E	*Energy*	8.2 keV
$\lambda = hc/E$	*Wavelength*	1.51 Å
2θ	*Scattering angle*	27.8°
$Q = (4\pi/\lambda)sin\theta$	*Momentum transfer*	2.0 $Å^{-1}$
$\Delta E/E$	*Energy resolution Si(111)*	$1.4 \cdot 10^{-4}$
s_v, s_h	*Vertical and horizontal beam size*	2 μm
d	*Sample thickness (droplet size)*	100 μm
p	*Pixel size*	50 μm
n_{pix}	*Number of pixels*	$352 \times 384 \times 2$
L	*Sample-detector distance*	1.3 m
w	*Speckle size*	≈100 μm
M_{rad}	*Radial resolution*	*10.81*
M_{det}	*Detector resolution*	*1.15*
$\tilde{\beta}_0$	*Analytically estimated contrast*	0.081

Supplementary Note 8: Thermal velocity model

Often the motion in the sub-100fs regime is attributed to thermal ballistic-like motion. This can be modelled by a Gaussian of the form:

$$F(Q,t) = F_0 \cdot e^{-[t/\tau(Q)]^2}$$

which corresponds to ballistic motion, harmonic oscillators and diffusion in a harmonic potential[13]. The time constant $\tau(Q) = (v \cdot Q)^{-1}$ in the ideal gas case can be related to the thermal velocity $v(T) = \sqrt{\frac{2k_B T}{m}}$, which corresponds to the most probable velocity of the distribution. Here, m is here selected to correspond to the mass of the water molecule and the k_B is the Boltzmann constant. The calculated curves are shown in Supplementary Fig. 8 for different temperatures *T.* For further comparison, the velocities are obtained analytically from the Maxwell-Boltzmann distribution:

$$P(v) = \sqrt{\left(\frac{m}{2\pi k_b T}\right)^3} 4\pi v^2 e^{-\frac{mv^2}{2k_B T}} \qquad (5)$$

This distribution is used to describe freely-moving particles in ideal gas, interacting only with brief collisions. In Supplementary Fig. 8 is shown the comparison between the Gaussian model and the Maxwell-Boltzmann, which exhibit identical velocities ranging from 4.8 Å/ps to 5.5 Å/ps in the temperature range 250K to 330K.

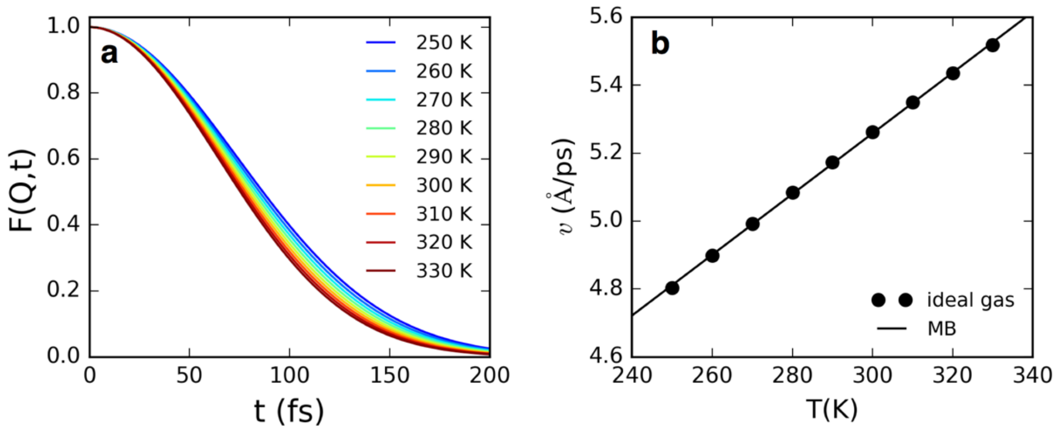

Supplementary Figure 8 | The thermal velocity model (a) The intermediate scattering function for different temperatures assuming a Gaussian form and thermal velocity. (b) The velocity obtained from the ideal gas approach and that from the Maxwell Boltzmann distribution.

Supplementary References

1. Chapman, H. N., Caleman, C. & Timneanu, N. Diffraction before destruction. *Phil Trans R Soc B* **369,** 20130313 (2014).
2. Herrmann, S. *et al.* CSPAD-140k: A versatile detector for LCLS experiments. *Nucl. Instrum. Methods Phys. Res. Sect. Accel. Spectrometers Detect. Assoc. Equip.* **718,** 550–553 (2013).
3. Sellberg, J. A. *et al.* Ultrafast X-ray probing of water structure below the homogeneous ice nucleation temperature. *Nature* **510,** 381–384 (2014).
4. Laksmono, H. *et al.* Anomalous Behavior of the Homogeneous Ice Nucleation Rate in "No-Man's Land". *J. Phys. Chem. Lett.* **6,** 2826–2832 (2015).
5. Kim, K. H. *et al.* Temperature-Independent Nuclear Quantum Effects on the Structure of Water. *Phys. Rev. Lett.* **119,** 075502 (2017).
6. Ding, Y. *et al.* Femtosecond x-ray pulse temporal characterization in free-electron lasers using a transverse deflector. *Phys. Rev. Spec. Top. - Accel. Beams* **14,** 120701 (2011).
7. Sikorski, M. *et al.* Application of an ePix100 detector for coherent scattering using a hard X-ray free-electron laser. *J. Synchrotron Radiat.* **23,** 1171–1179 (2016).
8. Hruszkewycz, S. O. *et al.* High Contrast X-ray Speckle from Atomic-Scale Order in Liquids and Glasses. *Phys. Rev. Lett.* **109,** 185502 (2012).
9. Lee, S. *et al.* Single shot speckle and coherence analysis of the hard X-ray free electron laser LCLS. *Opt. Express* **21,** 24647–24664 (2013).
10. Hruszkewycz, S. O. *et al.* High Contrast X-ray Speckle from Atomic-Scale Order in Liquids and Glasses. *Phys. Rev. Lett.* **109,** 185502 (2012).
11. Lehmkühler, F. *et al.* Single Shot Coherence Properties of the Free-Electron Laser SACLA in the Hard X-ray Regime. *Sci. Rep.* **4,** 5234 (2014).
12. Lehmkühler, F. *et al.* Sequential Single Shot X-ray Photon Correlation Spectroscopy at the SACLA Free Electron Laser. *Sci. Rep.* **5,** 17193 (2015).
13. Vineyard, G. H. Scattering of Slow Neutrons by a Liquid. *Phys. Rev.* **110,** 999 (1958).
